# A Folding Pathway Model of Mini-Protein BBA5

**DOI:** 10.1155/2015/828095

**Published:** 2015-09-20

**Authors:** In-Ho Lee, Seung-Yeon Kim, Jooyoung Lee

**Affiliations:** ^1^Korea Research Institute of Standards and Science, Daejeon 305-340, Republic of Korea; ^2^School of Liberal Arts and Sciences, Korea National University of Transportation, Chungju 380-702, Republic of Korea; ^3^Korea Institute for Advanced Study, Seoul 130-722, Republic of Korea

## Abstract

We present the folding pathway model of mini-protein BBA5, a bundle of secondary structures, *α*-helix and *β*-hairpin, by using action-derived molecular dynamics (ADMD) simulations. From ten independent ADMD simulations, we extracted common features of the folding pathway of BBA5, from which we found that the early stage chain compaction was followed by the formation of C-terminal *α*-helix. The N-terminal *β*-hairpin was observed to form only after *α*-helix was stabilized. This result is in good agreement with the experimental observation that BBA5 mutants were moderately cooperative folders, and their C-terminal helical fragments were of higher secondary structure propensity while the N-terminal hairpin fragments were of a random coil spectrum. We found that the most flexible part of BBA5 is the N-terminal four residues. Although both are made of the identical *ββα* motif, the secondary structure formation sequence of BBA5 is found to be different from that of FSD-1. Finally, a description of the folding pathway in terms of principal component analysis is presented to characterize the folding dynamics in reduced dimensions. With only three principal components, we were able to describe 83.4% of the pathway.

## 1. Introduction

Protein folding problem [1] is difficult to study experimentally and computationally. To become a functional protein, in many cases, it is necessary that the protein folds into its appropriate native structure. The folded native structure of a protein typically contains what is called the regular secondary structure (*α*-helix and *β*-strand). One important question to ask in studying the kinetic folding process of a protein is the temporal order of the formation of a native structure between, for example, the N-terminal part and the C-terminal part.

Depending on the order of the formation of the regular secondary structure of a protein and the compaction of the polypeptide chain, there are two views of the folding mechanism. In the diffusion-collision model [2] or the framework model [3], the regular secondary structure of a protein folds first and the folded secondary structure elements move around and collide with each other before they finally fold into the native structure. On the other hand, in the hydrophobic collapse model [4], the compaction of the protein occurs first and the formation of the regular secondary structure and tertiary structure progresses concurrently. All of these views were motivated to provide logical explanation that a typical protein molecule should fold into its native structure much faster than the age of universe as properly argued in the Levinthal paradox [5].

The regular secondary structure of a protein is formally defined by backbone hydrogen bonds between backbone amide hydrogens and carboxyl oxygens. The regular secondary structure (*α*-helices and *β*-sheet) can fold fast by local energy stabilization of hydrogen bonds. Typical energy gain per a hydrogen bond ranges from 3 to 7 kcal/mol, which is larger than the typical van der Waals interaction. Similarly, compaction of a protein assisted by solvation effect can happen at a much faster timescale than the folding time. Either through the fast formation of local regular secondary structures or by compaction via hydrophobic collapse, the number of relevant degrees of freedom is greatly reduced and protein folding can happen at a much faster timescale than the age of universe.

The mini-protein BBA5 (Ace-YRVpS YDFSR SDELA KLLRQ HAG-NH_2_; PDB code: 1t8j) can serve as a good model molecule to study the protein folding process via simulation. It is small, yet it contains both *α*-helix and *β*-hairpin. Struthers et al. [6, 7] experimentally designed this 23-residue protein in an iterative fashion so that it can adopt *ββα* motif regardless of the zinc binding. The D-proline residue at position 4 (shown by *p* in the sequence above) was essential in stabilizing *β*-hairpin in BBA5.

There are several folding studies of BBA5 and its mutants using molecular dynamics (MD) simulations and experiments. Snow et al. showed by simulation and temperature-jump experiments that BBA5 mutants are moderately cooperative folders [8].

Using an explicit solvation model, Rhee et al. [9] obtained multiple folding trajectories of BBA5 utilizing the Folding@Home method, which used a massively distributed computing strategy [9]. However, they had to modify potential energy parameters [10] of the AMBER94 all-atom force field [11] in order to obtain good agreement with experimental data over the original force field. Independent formation of secondary structure elements, helix and *β*-hairpin, was observed throughout the protein folding process of BBA5. The overall folding results agreed with those of Snow et al. [8] who used implicit solvation models for their simulations. The BBA5 folding was shown to be consistent with the diffusion-collision model, where secondary structural elements form independently and then collide to each other before finally forming the native structure.

Wang et al. [12] performed high-temperature unfolding simulation of BBA5 mutants. They found that the unfolding occurred through the disruption of the hydrophobic core and the unfolding of the helix. *β*-hairpin remained stable in the unfolding simulation owing to its stability associated with the turn connecting two *β*-strands. However, it should be noted that, generally speaking, the argument for the time-reversal symmetry often used to justify unfolding simulation of a protein to study its folding may not be valid with high-temperature nonequilibrium systems. We also note that, in a coarse-grained computational study of BBA5 [13], the importance of keeping chemical details was demonstrated when studying the kinetic mechanism of a small protein like BBA5.

From their MD simulation of BBA5, Jang et al. [14] found that misfolded protein structures were excessively stabilized. These were mainly due to the fact that salt bridges between ionic side-chains were stabilized excessively and the GBSA solvation potential preferred the helical propensity of the protein. Based on these observations, the solvation potential was modified, and the free energy contour map was calculated via MD simulation [14].

As mentioned, authors who studied the folding of BBA5 in terms of MD simulations had to modify their potential energy functions to better explain the folding mechanism of BBA5. However, it is not ideal that one needs to modify the force field used for the simulation of BBA5, and it is not obvious if the modified energy functions for BBA5 would work equally well for other protein molecules. We acknowledge that it is extremely difficult to fold a protein molecule properly by solving an initial value problem, that is, by starting with an initial conformation of the molecule and patiently waiting to observe it to fold into its native conformation using the standard MD simulation approach. The origin of this difficulty lies in the fact that the potential energy function used for the simulation may not be as accurate as it should be, so that the lowest-free-energy basin may not correspond to the true native basin.

To alleviate this difficulty, in this study, we considered utilizing the theoretical framework of solving a boundary value problem, where one is given with two-end states and one is asked to find the most optimal pathway to connect them. In particular, we used the action-derived molecular dynamics (ADMD) method [15–17]. In this approach, the input is two-end conformations (initial and final), and the output is a set of numerous all-atom conformations (i.e., a transition trajectory) that smoothly connects the two-end conformations. According to the physics of classical mechanics, the principle of stationary action is a variational principle that can be used to obtain the dynamics of a physical system. In ADMD, the most probable transition trajectory connecting two given boundary states is generated by minimizing the action of the system (see Section 2 for more details).

Previously, we have applied ADMD to study folding pathways of *α*-helix (acetyl-(Ala)_10_-N-methyl amide) and *β*-hairpin (residues 41–56 of protein G) using an empirical force field, where calculated dynamic folding pathway models were consistent with NMR experimental data reproducing the temporal sequence of backbone hydrogen-bond formation [18].

When the ADMD method was applied to study the folding pathway of 36-residue villin headpiece subdomain HP-36 [19], we found that the folding was initiated by hydrophobic collapse, after which concurrent formation of full tertiary structure and *α*-helical secondary structure was observed. The C-terminal helix was observed to form first, followed by the N-terminal helix positioned in its native orientation. The short middle helix was shown to form last [19]. We also carried out the ADMD simulation of FSD-1, *ββα*-motif (PDB code: 1fsd)/ [20], which is similar to BBA5 in the secondary structure composition. Comparison and discussions on the folding mechanisms of FSD-1 and BBA5 are presented later.

The remainder of the paper is organized as follows. In the following section, we describe the formalism of the ADMD method used to obtain the most probable pathway connecting between a fully extended structure and the native structure. In the next section, we present the ADMD results by analyzing obtained folding trajectories of BBA5. Finally, we conclude with a summary of the current work.

## 2. Computational Methods

The ADMD approach is quite different from the conventional MD approach. For a given temperature and a given initial state, conventional MD approach simulates conformational changes of a system fulfilling the Newton dynamics by solving an initial value problem. On the other hand, in ADMD, we solve a boundary value problem. That is, for two given states, we aim to obtain the most probable pathway smoothly connecting the two states, which approximately satisfies the Newtonian path conditions with a large-interval time step [15–17].

We divide the time interval [0, *τ*] into *P* slices with a time increment of Δ = *τ*/*P*. Then, the time at step *j* can be expressed as *t*_*j*_ = *j*Δ with *j* = 0,1, 2,…, *P*. The path {**q**_*j*_} is a collection of sequential structural frames with fixed initial **q**_0_ and final **q**_*P*_. The discretized action can be written as

(1) $$\begin{array}{c} S=\overset{P-1}{\underset{j=0}{\sum }}L_{j}\left(\left\{q_{j}\right\}\right)\Delta , \end{array}$$

where the discretized Lagrangian of *j*th temporal frame is defined as

(2) $$\begin{array}{c} L_{j}\left(\left\{q_{j}\right\}\right)=\overset{N}{\underset{I=1}{\sum }}\frac{m_{I}}{2\Delta^{2}}{\left(q_{I,j}-q_{I,j+1}\right)}^{2}-V\left(\left\{q_{j}\right\}\right). \end{array}$$

Here, the first term corresponds to the kinetic energy and *V*({**q**_*j*_}) is the potential energy. *N* is the total number of atoms, *m*_*I*_ is the mass of *I*th atom, and **q**_*I*,*j*_ is the position vector of *I*th atom at *j*th frame. The stationarity condition, *δS* = 0, leads to a set of linear equations.

Rather than concentrating on the integration of the equations of motion, we examine various transition pathways by minimizing the classical action of (1) above with as much low potential energy as possible. As in our previous ADMD studies, we used the AMBER94 all-atom force field [11] with GBSA solvation model [21] as implemented in the TINKER package [22].

Recently, a number of closely related methods that are similar in spirit to ADMD were introduced [23–25]. For more details of the formalisms of these methods, readers are referred to [15–20].

Since we study the folding of BBA5, one obvious end structure is the native structure of BBA5. In practice, we used an energy-minimized structure starting from the native structure. The backbone RMSD (root-mean-square deviation) between these two structures is 0.75 Å. There are many possibilities to assign for the other end structure, and we choose to use an energy-minimized structure staring from the fully extended structure of BBA5 for it. Now, these two energy-minimized end structures are treated as the “reactant” (extended structure) and the “product” (native structure), and we search for transition pathways that smoothly connect them following the Newton dynamics.

Our primary goal of this study is to delineate, at atomic resolution, the global folding sequence of BBA5 by obtaining the lowest-potential-energy-barrier pathway between the two-end states. In this work, we refer to the trajectory with the lowest-potential-energy barrier as the most probable transition pathway model. At the beginning of the ADMD simulation [15–17], a set of random numbers was generated to construct a trial atomic trajectory for each atom of BBA5 in a random fashion. The number of atomic conformations in order to smoothly connect two-end conformations of BBA5 was set to 1999, so that *j* = 0 and *j* = 2000 conformations, respectively, correspond to the extended structure and the native structure. As a total, we dealt with 2001 conformations for the ADMD simulation of BBA5, and we found that 2001 was a large enough number to connect the “reactant” and “product” conformations smoothly. We note that both of the atomic conformations, at step indices *j* = 0 and *j* = 2000, are relaxed through a local energy minimization procedure. Starting from a locally stable extended BBA5 conformation (*j* = 0), we obtained a series of molecular conformations, which dynamically connects to the native conformation (*j* = 2000) in a smooth fashion.

One of critical technical problems in the protein folding simulation is the accuracy of the potential energy function used, which is beyond the scope of this work. In a conventional molecular dynamics simulation, often, the folding event is hard to observe due to this problem. In most folding studies of a protein, there is no guarantee that the native state of the protein is the lowest-free-energy state of a given potential energy function, and otherwise obviously the folding problem would have been already solved. In the ADMD formalism, we seek to find what one can do even when it is not known if the native state of the protein is the lowest-free-energy state of a given potential energy function by asking the question of obtaining the most probable pathway out of all possible paths connecting two-end states. We note that the folded state of BBA5 used in this work is a locally stable state since this is an energy-minimized state from the native structure of BBA5.

**Figure 1:**
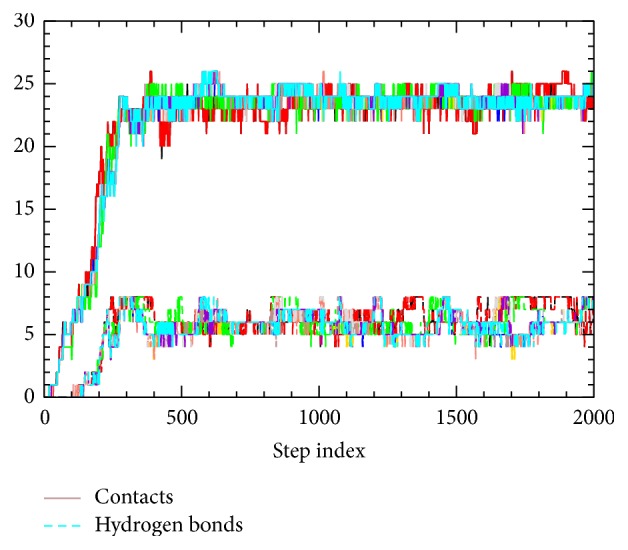
The dynamic variation of the tertiary contact formation and the hydrogen-bond formation of all ten pathways performed in this work is shown. The tertiary contact formation is represented by the numbers of native contacts (shown in solid lines located near 25) and the hydrogen-bond formation by the number of hydrogen bonds (shown in dashed lines near 8) being shown along the ADMD step index.

In order to obtain the most probable transition pathway, we searched for low-potential-energy-barrier pathways. In practice, a total of 10 independent ADMD simulations were carried out to obtain 10 low-potential-energy-barrier pathways, and we seek to find common features among the 10 generated pathway models. In Figure 1, we provide data for the dynamic variation of the tertiary contact formation and the hydrogen-bond formation of 10 pathways. By visual inspection, we find that the 10 pathways are all quite similar to each other forming one cluster. This should be contrasted to the case of FSD-1 [20], where three clusters were identified out of 10 pathways. Since there were no noticeable differences among the 10 pathways, we picked the lowest-potential-energy-barrier trajectory among them, and in this work all data shown subsequently correspond to this trajectory.

The advantages of ADMD protein folding simulation lie in its ability to secure a reasonable degree of uniformity in serial collection of the meaningful conformations related to the folding event. The method effectively filters short- and intermediate-range timescale (<Δ and ~Δ) fluctuations/motions of the molecule under investigation. The ADMD simulation can be performed in a highly parallel fashion, allowing us to carry out a formidable scientific challenge without getting trapped in local minima on rugged energy landscapes. Although the ADMD method has an advantage over other methods in sampling long-time trajectories, its reliability when studying a hard problem such as the protein folding event may depend on the adequacy of the potential energy function used.

## 3. Results and Discussion

### 3.1. Hydrophobic Collapse

**Figure 2:**
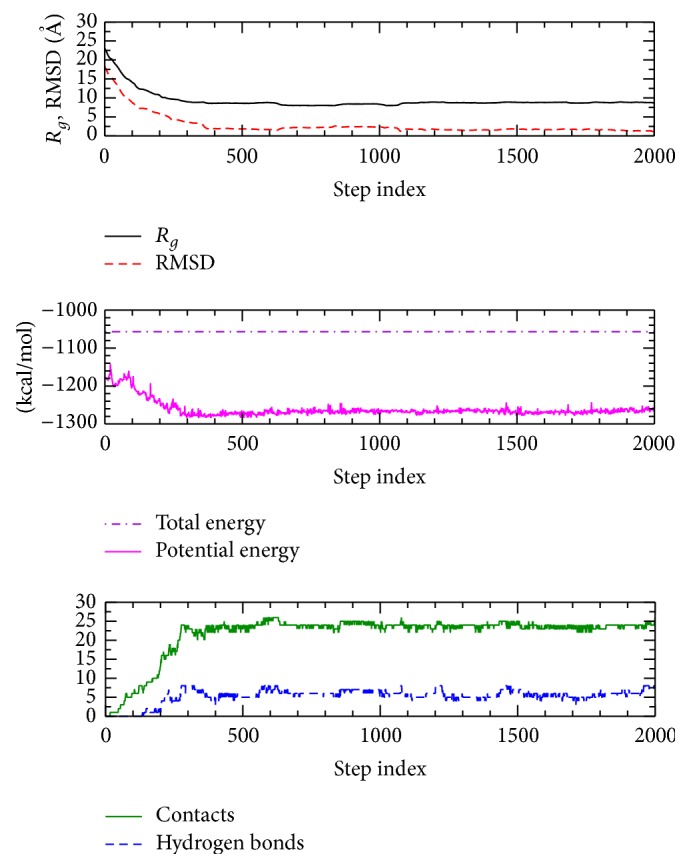
From the lowest-potential-energy-barrier pathway out of 10 runs, we show the radius of gyration (*R*_*g*_), the root-mean-square deviation (RMSD) from the final native structure, the total energy, the potential energy, and the numbers of native contacts and hydrogen bonds for BBA5, as a function of the time step index. A sharp change of the RMSD values in the early stage of the folding process is clearly shown in the figure. Other quantities also show similar behaviors along the folding pathways. Total energy conservation is obtained via ADMD simulation. The first extended conformation (*j* = 0) and the last native conformation (*j* = 2000) are shown in Figure 3.

In Figure 2, various structural properties are shown along the folding pathway of BBA5. In the top panel, the RMSD value calculated from the local-energy-minimized native conformation of BBA5 and the radius of gyration (*R*_*g*_) of BBA5 are shown to decrease monotonically till 500th step. During the early stage of folding, *R*_*g*_ decreases drastically from its initial value of 23.0 Å to about 8.6 Å, representing a rapid compaction of the chain. This overall chain compaction is well represented by the decrease of other quantities shown in Figure 2, potential energy, and RMSD. RMSD and potential energy are also highly correlated with the correlation coefficient of 0.903.

The numbers of native contacts and native backbone hydrogen bonds are shown in the bottom panel of Figure 2. As stated above, the corresponding variation of this figure arising from all 10 ADMD pathways is shown in Figure 1. A native contact is defined to exist between two residues, which are separated by more than two residues in sequence, if their C^*α*^-C^*α*^ distance is less than 6.5 Å. A backbone native hydrogen bond is defined to exist if the distance between a backbone carboxyl oxygen and a backbone nitrogen is separated by less than 2.5 Å and the bending angle from oxygen-hydrogen-nitrogen is greater than 135°. Like other structural variables shown in Figure 2, similar variations along the folding pathway are observed.

**Figure 3:**
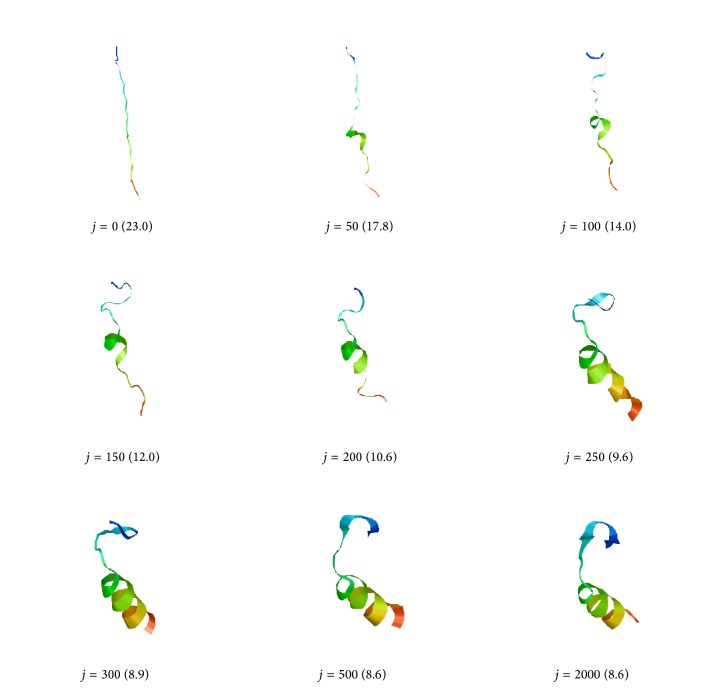
Snapshots of the lowest-potential-energy-barrier pathway out of 10 BBA5 ADMD simulations are shown. The initial (final) conformation shown at *j* = 0 (2000) is prepared by applying local energy minimization to the fully extended (NMR native) structure of BBA5. The color variation of the structure from blue to red corresponds to its amino acid sequence variation from N- to C-terminus. Numbers in parentheses represent the values of radius of gyration in Å.

Snapshots of the lowest-potential-energy-barrier pathway out of 10 BBA5 ADMD simulations are shown in Figure 3. Numbers in parentheses represent the radii of gyration in Å for the structures shown. The initial (final) conformation shown at *j* = 0 (2000) was prepared by applying local energy minimization to the fully extended (NMR native) structure. ADMD simulations were conducted using the AMBER94 all-atom force field with the GBSA solvation model. The color variation from blue to red corresponds to the amino acid sequence variation of the polypeptide chain from N- to C-terminus.

**Figure 4:**
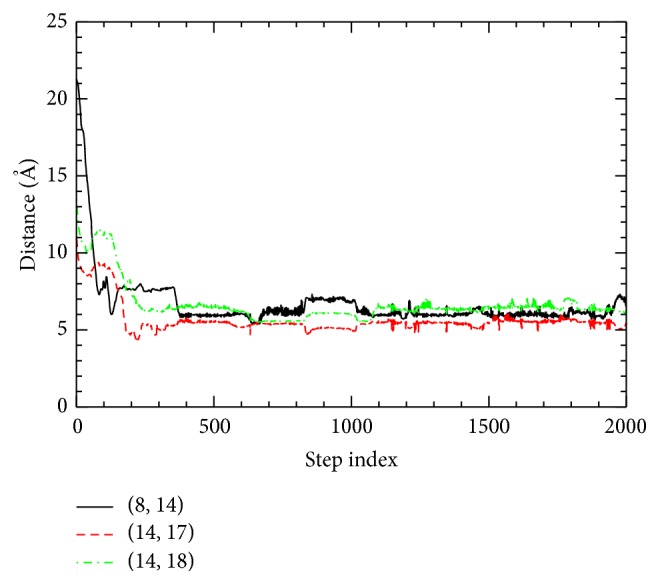
C^*α*^-C^*α*^ distances associated with contacts (8, 14), (14, 17), and (14, 18) are plotted along the folding pathway. *x*-axis shows the ADMD step index along the trajectory.

In the BBA5 sequence, there are four hydrophobic residues, F8, L14, L17, and L18, forming the hydrophobic core of the protein. To understand the formation of this core along the folding pathway, we measured the three native contacts [(8, 14), (14, 17), and (14, 18)] among the four residues and show them in Figure 4. C^*α*^-C^*α*^ distance associated with the contact (8, 14) changes from 21.31 Å to 6.49 Å during the folding pathway, where its 90% reduction occurs around the step index of *j* = 74 in Figure 4. At *j* = 74, a total of six contacts of (3, 6), (10, 13), (11, 14), (12, 15), (12, 16), and (13, 16) are formed, while no native backbone hydrogen bonds are formed. We note that the contact (3, 6) belonging to *β*-hairpin region fluctuates on and off during early stages of the folding process.

By measuring the number of contacts and that of backbone hydrogen bonds together, one can investigate the rates of tertiary structure formation (i.e., protein folding) and the formation of secondary structure elements. The strong correlation between *R*_*g*_ and RMSD is not particularly interesting especially with folding events of small proteins. However, it is interesting that, in the case of BBA5 folding, the correlation between *R*_*g*_ and the number of native contacts is rather strong. When 70% of *R*_*g*_ was reduced to the native value (23.0 Å→12.9 Å) along the folding pathway, ~12.5% of native backbone hydrogen bonds and ~30.7% of native contacts were formed. This means that the hydrophobic collapse of BBA5 is a relatively early event, and it occurs well before the considerable formation of either secondary structures or native contacts. In addition, the majority of the potential-energy reduction was not yet observed at the time of significant hydrophobic collapse (70% of *R*_*g*_ reduction) process (i.e., *j* < 142). According to the diffusion-collision model or the framework model of protein folding, before a protein structure is collapsed to its compact size, a significant amount (more than 50%) of secondary structure formation is anticipated [2], while according to the hydrophobic collapse model [4] hydrophobic collapse happens before the formation of secondary structures or tertiary structure. The current folding pathway for BBA5 is more consistent with the hydrophobic collapse model [4] than the diffusion-collision model [2]. One of the important differences between the hydrophobic collapse model and the diffusion-collision model is the sequence of the structure formation (secondary structure versus tertiary structure). In our ADMD simulation of BBA5, we observed the simultaneous formation of the secondary structure and the tertiary structure (see Figures 2 (bottom panel), 4, 5, and 6 for step indices between 100 and 400), which led to our conclusion that our model for BBA5 is consistent with the hydrophobic collapse model.

We note that the folding model of BBA5 by Rhee et al. [9, 10] which is consistent with the diffusion-collision model and the current model which is consistent with the hydrophobic collapse model are both consistent with the observed secondary structure propensity by the laser temperature-jump experiments of Snow et al. [8]. However, Rhee et al. had to modify their potential energy parameters to obtain the secondary structure propensity and no such modification was performed in this study. To determine which of the two folding models is more realistic is beyond the scope of this study, and this can be probably decided by ingenious experimental setups in future.

### 3.2. The Order of the Secondary Structure Formation

**Figure 5:**
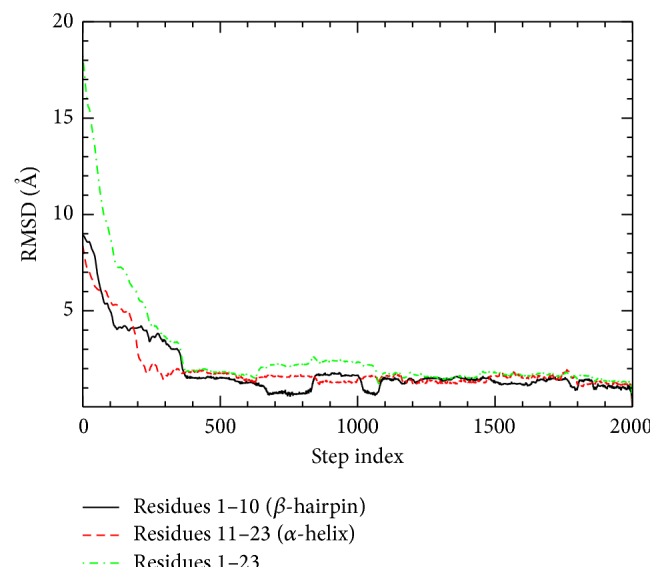
Local RMSD values (residues 1–10 and 11–23) and the overall RMSD value (residues 1–23) are plotted along the folding pathway. The RMSD values with respect to the native structure are plotted.

As a more specific analysis of a folding event, one can consider both local and global structure variations together along the folding pathway. Local RMSDs measured for N- and C-terminal parts are shown in Figure 5 along with the RMSD of the whole chain.

With this definition one can observe the respective local relaxations of the secondary structure components. The steady decrease of the RMSD value along the folding pathway (150 < *j* < 400) is consistent with the potential-energy decrease. From the local RMSD of *β*-hairpin, we observe that it decreases rather slowly after it reaches to about 4 Å (120 < *j* < 360). On the contrary to this, the local RMSD of *α*-helix decreases much faster before it reaches to near its final value (*j* < 200). This is due to the fact that *α*-helix can form through local atomic movements in terms of sequence separation, not much associated with nonlocal atomic arrangement. In all of the 10 ADMD simulations, the C-terminal *α*-helix is observed to form first, which is followed by *β*-hairpin formation. Thus, the current BBA5 folding model does not agree with concurrent or independent formation of the secondary structure along the folding pathway. Our folding pathway obtained by ADMD is consistent with the results of Snow et al. Based on simulations and laser temperature-jump experiments [8], Snow et al. showed that BBA5 mutants are moderately cooperative folders, and the C-terminal helical fragment was of higher secondary structure propensity while the N-terminal hairpin fragment was of a random coil spectrum [7, 8].

### 3.3. The Order of Contact Formation

**Figure 6:**
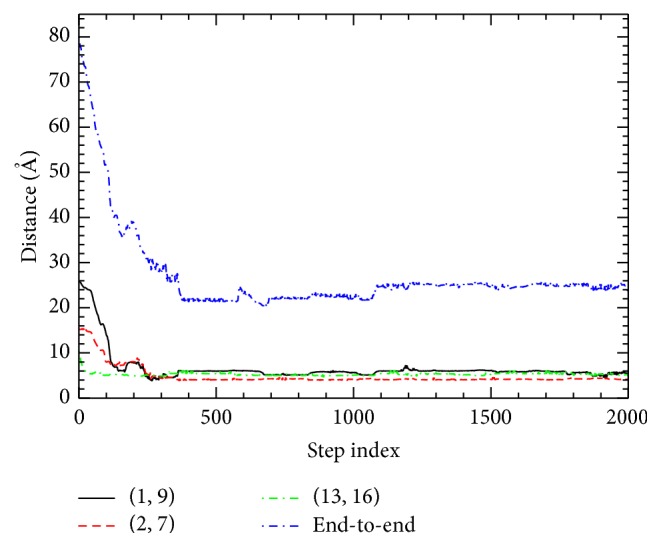
C^*α*^-C^*α*^ distances are plotted along the folding pathway. End-to-end distance is plotted along the folding pathway. Contacts (2, 7) and (13, 16) correspond to the charged amino acid pairs (R:+, D:−) and (E:−, K:+), respectively.

The orders of the contact and backbone hydrogen-bond formations of BBA5 are examined. The sequence of the contact formations in the early stage of the folding is as follows: (13,16)→(12,15)→(11,14)→(10,13)→(12,16)→(3,7). Similarly, the sequence of the backbone hydrogen-bond formations in the early stage of the folding is as follows: (12,16)→(13,17)→(15,19)→(16,20). In Figure 6, to observe both *β*-hairpin formation and overall folding process from the current folding model, C^*α*^-C^*α*^ distances corresponding to the contacts of (1, 9) and (2, 7) are shown. Although calculated distances (7.81 Å and 8.05 Å) are greater than 6.5 Å at *j* = 200, the local folding of *β*-hairpin is about to occur at this stage. We observe that, at *j* = 200, 13 residue-residue contacts and 2 backbone hydrogen bonds are already formed in the current folding model. Two contacts of (2, 7) and (13, 16) are between oppositely charged amino acid residue pairs (R:+, D:−) and (E:−, K:+). These salt-bridge interactions play important roles in the initial compaction of the molecule. The contact (13, 16) is the first native contact formed in the current folding model. This is due to the short sequence separation between opposite charges. These data all represent again that, during the folding process of BBA5, the C-terminal *α*-helix forms first, which is followed by *β*-hairpin formation. We find that secondary structure begins to form only after the hydrophobic core and some initial tertiary contacts are established. The hydrophobic collapse is observed by the rapid reduction of the radius of gyration in the initial stage of folding.

### 3.4. BBA5 versus FSD-1

Recently, we carried out all-atom ADMD folding simulations of the full-size FSD-1 [20]. FSD-1 is another designed mini-protein with 28 residues containing both *α* and *β* secondary structure elements [26, 27]. From the ADMD simulation of FSD-1, we observed multiple folding pathways in contrast to the case of BBA5. This was consistent with existing computational studies [20]. Hydrophobic collapse was observed first, and then subsequent folding events proceeded by forming either *α*-helix or *β*-hairpin. The folding pathway of FSD-1 elucidated by ADMD simulations did not follow the scenario of the framework model. Experimental data indicated that the folding of FSD-1 was weakly cooperative [26]. Understanding the folding behaviors of the two mini-proteins (BBA5 and FSD-1) with the identical *ββα* motif serves as a stepping stone towards the ultimate understanding of the general protein folding mechanism.

### 3.5. Principal Component Analysis

Principal component analysis (PCA) [28] is a mathematical tool to analyze correlations from a large set of data. In practice, it can be used to reduce dimensionality by extracting a small number of most contributing elements (principal components) from the data. Here, we define the covariance matrix *M* of the spatial fluctuation as

(3) $$\begin{array}{c} M_{ij}=\langle \left(x_{i}-\langle x_{i}\rangle \right)\left(x_{j}-\langle x_{j}\rangle \right)\rangle , \end{array}$$

where *x*_1_, *x*_2_, *x*_3_,…, *x*_3*N*_ are the Cartesian coordinates of *N*  C^*α*^ atoms. The average 〈⋯〉 is taken over all structural frames from the ADMD trajectory (i.e., *P* + 1 = 2001). The matrix *M* contains information on the spatial correlation between all residue pairs. Usefulness of PCA should be validated by a significant amount of coverage of *M* with only a small number of principal components. By diagonalizing the covariance matrix *M* of size 3*N* × 3*N*, 3*N* eigenvectors with their corresponding eigenvalues are obtained.

**Figure 7:**
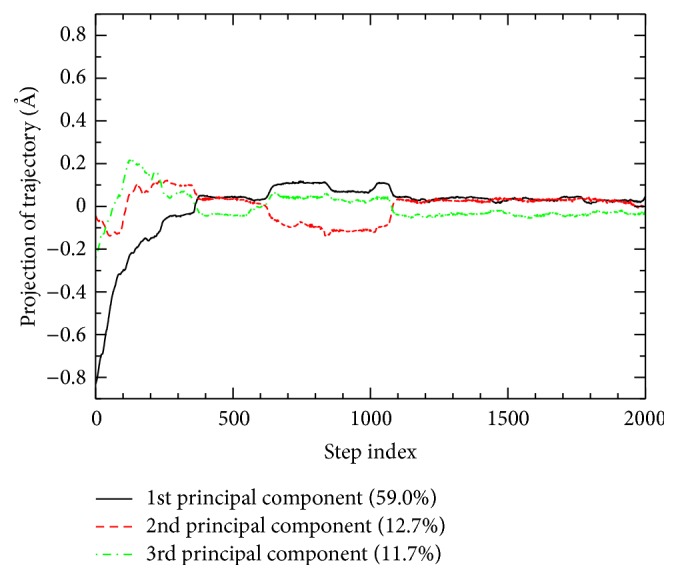
The folding pathway is projected to the first three principal components, which are responsible for a total of 83.4% of the covariance matrix.

**Figure 8:**
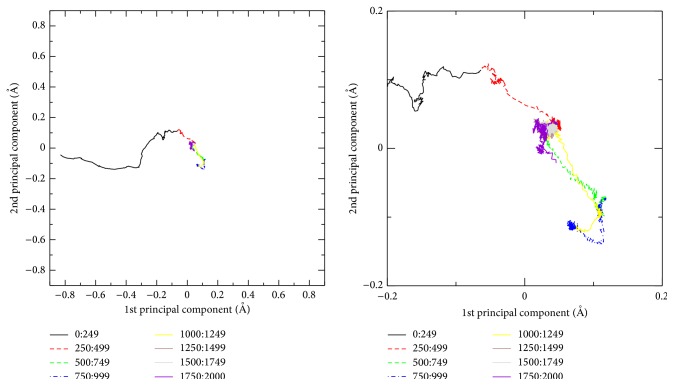
The folding trajectory is projected into the first two principal components. These two components account for about two-thirds of the variance matrix. ADMD pathway steps are indicated by colors.

Figure 7 shows the projections of the folding pathway onto the eigenvectors corresponding to the three largest eigenvalues of *M*. These principal components can serve to describe the folding event in terms of 83.4% of total fluctuations. The PCA projections [28] of the protein folding pathway onto two-dimensional plot characterized by the two largest-variance principal components are shown in Figure 8. The first two components account for over 71% of total variance allowing us to grasp the majority of the folding pathway information to be displayed in two dimensions. Eight equal ADMD step intervals are indicated in the folding pathway with various colors. As shown in the figure, the early stage (0 ≤ *j* ≤ 249) exhibits a rapid change.

**Figure 9:**
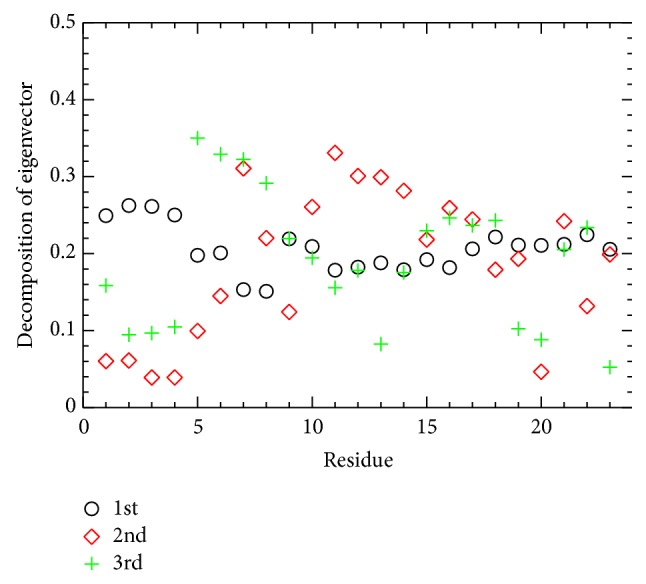
C^*α*^ atom contribution to the first three principal components is shown as a function of residue index. *y*-axis corresponds to the magnitude of three components (*x*, *y*, and *z*) contributing to the corresponding principal component.

The degree of atomic fluctuations is measured for each residue.  Figure 9 shows the residue-level contribution to the principal components shown, indicating the protein folding process approximately. The contribution to the atomic fluctuation during the folding process is identified once again through the PCA method. As far as the first principal component is concerned, the N-terminal part is more flexible than the middle part of the protein. In this work, the most flexible region of BBA5 is the N-terminal loop, which is in good agreement with circular dichroism spectra [8]. The contacts involved in *β*-hairpin are more difficult to form than *α*-helix ones because strands aligned are usually distantly located in sequence as in (1, 7), (1, 8), (1, 9), and (2, 7), found in BBA5.

This has to do with the experimental observation of the circular dichroism spectra. The current analysis shows that the N-terminal hairpin (1–10) is in a random coil spectrum [8] in the early stage of folding. This compares with the experimental observation that the C-terminal helices (11–23) are of higher secondary structure propensity [8]. As mentioned before, the current folding model supports the earlier formation of *α*-helix compared to *β*-hairpin.

## 4. Conclusions

We have applied the ADMD method to the study of BBA5 folding. A total of 10 independent ADMD simulations were performed, from which we extracted common features for the transition pathway of BBA5. In the initial stage of the folding, overall size of the protein measured by *R*_*g*_ was drastically reduced, representing rapid compaction of the protein. The calculated folding pathway is more consistent with the hydrophobic collapse model than the diffusion-collision model, in which the concurrent formation of tertiary structure (native contacts) and secondary structure (*α*-helical content) is emphasized on equal footing after hydrophobic collapse. Stable secondary structures began to form only after the collapse, suggesting that BBA5 folding follows neither the framework model nor the diffusion-collision model. The most flexible parts of BBA5 were found to be the N-terminal four residues, in good agreement with experimental data [8].

After the initial hydrophobic collapse, the C-terminal *α*-helix formed first, which was followed by the N-terminal *β*-hairpin formation. This should be contrasted to the secondary structure formation sequence of FSD-1 [20], which contains the identical secondary structure motif of *ββα*, where multiple folding pathways [20] were observed with independent formation of *α*-helix and *β*-hairpin.

Finally, a description of the folding pathway in terms of the principal component analysis is presented to characterize the folding dynamics in reduced dimensions, where 83% of the pathway can be described by three principal components.
